# Target Validation: Linking Target and Chemical Properties to Desired Product Profile

**DOI:** 10.2174/156802611795429185

**Published:** 2011-05

**Authors:** Paul G Wyatt, Ian H Gilbert, Kevin D Read, Alan H Fairlamb

**Affiliations:** Drug Discovery Unit, College of Life Sciences, University of Dundee, Dundee, DD1 5EH, UK

**Keywords:** Chemical validation, druggability, drug resistance, genetic validation, neglected diseases, molecular targets, target assessment, target product profile.

## Abstract

The discovery of drugs is a lengthy, high-risk and expensive business taking at least 12 years and is estimated to cost upwards of US$800 million for each drug to be successfully approved for clinical use. Much of this cost is driven by the late phase clinical trials and therefore the ability to terminate early those projects destined to fail is paramount to prevent unwanted costs and wasted effort. Although neglected diseases drug discovery is driven more by unmet medical need rather than financial considerations, the need to minimise wasted money and resources is even more vital in this under-funded area. To ensure any drug discovery project is addressing the requirements of the patients and health care providers and delivering a benefit over existing therapies, the ideal attributes of a novel drug needs to be pre-defined by a set of criteria called a target product profile. Using a target product profile the drug discovery process, clinical study design, and compound characteristics can be defined all the way back through to the suitability or druggability of the intended biochemical target. Assessment and prioritisation of the most promising targets for entry into screening programmes is crucial for maximising chances of success.

## DRUGS FOR NEGLECTED TROPICAL DISEASES

One sixth of the world’s population - approximately 1 billion people - are infected with neglected tropical diseases (NTDs) including the vector-borne parasitic diseases, African sleeping sickness, Chagas disease, leishmaniasis, filariasis, onchocerciasis and schistosomiasis [1]. Although, the existing drugs used to treat many NTDs have serious limitations, only 1% of all new drugs to reach the market in the past 25 years were for neglected diseases [2]. Issues with current therapies include: cost; difficulties in administration; poor safety profile; and lack of efficacy e.g. due to drug resistance [3,4]. In total, NTDs account for 5% of the global disease burden; yet, it is estimated only about 0.1% of global research budgets are spent on drug discovery for these diseases. This lack of resource is compounded by the high attrition rates for drug discovery, as only 1 in 5 projects survives through preclinical development and less than 1 in 10 entering clinical development are finally registered for clinical use [5]. Most projects fail either through issues with the underpinning biology. For example, selected targets are often subsequently revealed as not essential for parasite growth or survival and a multitude of potential pitfalls are encountered along the drug discovery pathway, including: failure to identify suitable drug-like lead compounds for optimization; lack of efficacy, often in clinical trials; toxicity and drug metabolism and pharmacokinetic (DMPK) issues.

Despite substantial research into the biology of these parasites and the sequencing of their genomes, discovery of candidate drugs is hampered by the lack of well validated druggable targets as many essential genes are not druggable. In addition the translation of genetic validation of targets into candidate compounds with potent anti-parasitic effects suffers from high rates of attrition. Therefore, there is a need for well validated and characterised targets, which can deliver drug candidates capable of addressing the Target Product Profiles (TPP), i.e. the desired properties and efficacy of the drug, for these diseases. In consequence, the purpose of this review is to illustrate how molecular targets are assessed for entry into a drug discovery pipeline, taking into account of needs of the patients as defined by the TPP. The authors hope this discussion will encourage researchers to generate the required data for target assessment as a matter of course during their research, thereby producing sufficient potential anti-parasitic drug targets to support future drug discovery efforts.

This review will focus on the molecular target approach to drug discovery and how the use of TPPs for individual diseases can help to accelerate the drug discovery process and help to reduce the high attrition rates seen during drug discovery and development. In addition, this approach should also be used to assess and develop compound series identified by *in vitro* screening against whole parasites, a valuable alternative strategy for anti-parasitic drug discovery [6-9].

## TARGET PRODUCT PROFILES

For drug projects to succeed in delivering the right medicine for the right patient there must be from the outset a clear understanding of the critical features of the final therapeutic product for clinical use. These features are defined in the Target Product Profile (TPP), which is a listing of the essential attributes required for a specific drug to be a clinically successful product and to be of substantial benefit over existing therapies.

The TPP is an important strategic planning and decision-making tool [10,11] that is used to define: the target patient population; acceptable levels of efficacy; acceptable levels of safety; the dosing route; the dosing schedule; the properties of the formulated drug; and acceptable levels of cost of the formulated drug. To compile a TPP it is essential that there is a good knowledge of the patient’s needs, so it is important to include information provided by health care workers and physicians, health regulators and policy makers, especially from disease endemic countries. In addition, the TPP needs to take into account the existing therapies and define those attributes required for the new therapy to present a significant advance over the current clinically used drugs. The TPP has to be periodically reassessed with regard to meeting essential, preferred or minimal acceptable criteria, as the unmet clinical need changes with the development of competing drug candidates and emerging data from clinical trials.

By way of example, two contrasting parasitic diseases TPPs are shown in Table **1**. This profile should be used as the basis for assessing project feasibility, monitoring progress and guiding drug discovery and development activities throughout the critical path for the entire research and development process. In this role the TPP provides the information necessary for: selection of targets; selection of screening hits and chemical start points for optimisation; the optimisation of drug candidates; objectivity for the project decision making process; better design of clinical trials; and open and constructive communication with the regulatory agencies.

The TPP can be used during the earlier phase of the drug discovery process to define the attributes required for drug development candidates or compound series to pass from one phase of the drug discovery process to the next. In fact, consideration of TPPs can be taken right back to the appraisal and selection of targets and hits from screening campaigns. Clearly defining a TPP and using it to define criteria for progression from one phase of the drug discovery process to another helps to deliver benefits from better decision making, faster development times and higher approval success rates to bring a drug to market. Most importantly this approach helps to define targets and compound series which will never achieve the TPP and therefore never benefit patients, facilitating the rapid closure of such projects and allowing the realignment of valuable resource into potentially more productive areas.

## MOLECULAR TARGET ASSESSMENT

In order to initiate a drug discovery programme, a pool of putative targets will require assessment to identify those which offer the best chance of delivering drug candidates to address the unmet medical need. The process thereafter is beset with potential pitfalls and high attrition seen in all drug discovery endeavours; however the chances of ultimate success can be increased by intelligence-based assessment and selection of these targets. This assessment needs to remain in the context of the TPP to ensure the unmet medical need is truly being addressed. This target assessment and prioritisation process is well established in the drug discovery industry, so this article will illustrate how this paradigm can be appropriately applied to assess targets to establish a parasitic disease discovery pipeline. The criteria of key importanceused to assess targets are described in Table **2**. A scoring system using the globally familiar colours of traffic lights is applied for each criterion. Scoring each target with the traffic light system affords an initial overall view of the pipeline under consideration, allowing a ranking of the portfolio, identifying the most mature targets and highlighting any key areas of weakness. In the suggested model, for most cases, a red assignment is not an absolute “stop”, but represents the current status of the target and highlights where further studies and/or reagents are required in order to advance a target towards readiness for entry into the drug discovery pipeline. It is important to note that some targets are more likely to fit certain TPPs than others. For example, targets that are predicted to be rapidly cidal following inhibition by small molecules are preferable to those that are slowly cidal or simply cytostatic, particularly if a fast response to drug treatment is specified by the TPP. This also has implications from a pharmacological perspective, since compounds that are cytostatic require prolonged and sustained exposure levels above the minimal inhibitory concentration to be curative. For example, the prolonged therapeutic schedule required for treatment of human African trypanosomiasis with the cytostatic drug, eflornithine [12] suggests that ornithine decarboxylase is not an ideal target for further drug discovery. There are also immunological implications, since cytostatic drugs require an effective host immune response in order to obtain cures. For example, the cidal drug, liposomal amphotericin B is the current treatment of choice for visceral leishmaniasis in cases co-infected with HIV [13].

## TARGET VALIDATION: TWO COMPLEMENTARY APPROACHES

With the wisdom of hindsight, failure of target-based drug discovery campaigns can often be attributed to inadequate scientific knowledge about the functional roles of a molecular target under physiological conditions inside and outside a cell. The two main complementary approaches to target validation can be categorised as “chemical” and “genetic”, where small molecule inhibitors or genetic methods can be used to modulate the functional activity of a target. Broadly speaking, the former provides chemical evidence for druggability of the target and favourable selective toxicity against the pathogen versus the host (cell, tissue or whole animal), while the latter provides genetic evidence of essentiality of function within the pathogen. Tools are available to achieve either target knockdown (e.g. reversible inhibitors or RNA interference) or target knockout (e.g. irreversible inhibitors or gene deletion). As summarised in Table **3** and discussed below, neither method on its own is sufficiently robust to provide absolute proof of essentiality and, whenever feasible, complementary and confirmatory evidence should be sought using both approaches.

Chemical validation involves the use of drugs or experimental compounds to provide evidence that specific inhibition of a target results in inhibition of growth or death of the parasite. Most parasites display complex life cycles between their vertebrate and invertebrate (vector) hosts and can modulate their metabolic activities in response to changes in the external environment (e.g. pH, temperature, nutrient availability). Thus, the strength of chemical validation increases in the following order: (i) *in vitro* activity against vector stages; (ii) *in vitro* activity against host stages; (iii) *in vivo* activity in animal models; and, best of all, (iv) therapeutic activity in patients. The major advantage of the chemical approach is that it addresses several key druggability issues: cell permeability and selective toxicity in whole cell assays *in vitro; *suitable drug metabolism and pharmacokinetic (DMPK) properties *in vitro* and *in vivo*; and acceptable safety and efficacy profile in appropriate animal models.

Chemical approaches also have the distinct advantage in being able to identify non-protein targets that could not be predicted from analysis of the genome. Classical examples are: detoxification of haem into haemozoin in malaria parasites, the target for 4-aminoquinolines such as chloroquine [14,15]; and the ergosterol-rich plasma membranes of leishmania, the target for amphotericin B [16,17]. Chemical methods can also uncover compounds (pro-drugs) that undergo bio-activation to highly toxic compounds (lethal synthesis). For example, trypanosomes are highly susceptible to nitro-heterocyclic compounds such as nifurtimox through metabolic activation by a number of mechanisms [18-21]. Likewise, leishmania, but not humans, can convert allopurinol into aminopyrazolopyrimidine ribonucleotides with subsequent incorporation into RNA [22]. Finally, some drugs are thought to act via “selective distribution” [23], where compounds are selectively concentrated by parasite transporters. The melaminophenyl class of arsenical drugs used to treat African sleeping sickness is a classic example of this mechanism [24-26]. None of these targets could be reliably predicted with the currently available tools to analyse parasite genomes.

However, there are weaknesses to the chemical validation approach. Highly specific inhibitors are usually not available at the outset of a discovery campaign. Consequently, structure-activity-relationships between target (IC_50_) and whole cell (EC_50_) inhibitory potency may be poor, due to lack of specificity or variable cellular pharmacokinetics. This is especially true for less specific generic inhibitors against target classes such as protein kinases and proteases. Demonstration that compounds are acting “on-target” can also be technically difficult. Moreover, identification of the target (or targets) for a small molecule inhibitor identified through a whole cell phenotypic screen can present significant challenges.

Genetic approaches to target validation can be broadly classified as target (gene) knockout and target (RNA) knockdown methodologies. If structural and mechanistic knowledge of the target is known, then a dominant-negative approach to target suppression can be employed in some situations [27]. A novel strategy, which may be more generally applicable, involves expression of a mutated FK506-binding protein fused to the N-terminus of the target. The stability of the fusion protein is dependent on binding of the ligand FK506 – removal of the ligand destabilises the FKBP domain resulting in protein degradation. To date the method has been successfully used in *P. falciparum* [28,29], *T.gondii* [30] and *L. major* [31]. However, it should be born in mind that addition of FKBP could interfere with correct assembly of protein complexes, enzyme function or subcellular targeting.

The particular methodology employed for genetic validation depends on the molecular toolbox available for any given organism (see reviews [32,33]). The available tools are limited by factors such as the availability of inducible or non-inducible expression vectors; the range of drug-selectable markers; and the genetic and physiological properties of the organism under study (e.g. gene copy number, ploidy; ease of culture in defined media; susceptibility to drug selection; ease of transfection; sexual recombination). Genetic manipulation of *Leishmania* and *Trypanosoma* [33,34] is generally easier than that of *Plasmodium* [35], although genetic manipulation of the related apicomplexan parasite, *Toxoplasma* *gondii* [36], can provide clues about probable outcomes in malaria.

The genetic approach of targeted gene disruption or gene replacement (gene knockout) is generally regarded as the most definitive method for target validation [37,38]. This method involves introduction into cells of a DNA construct carrying a selectable marker (typically a gene conferring resistance to a drug) flanked by 5'- and 3'-flanking sequences of the target gene of interest. Following transfection, homologous recombination occurs in some of the population such that the target gene is replaced with a drug resistance gene in the chromosome. Cells lacking this replacement are eliminated by selection with a toxic agent, such as G418, hygromycin or phleomycin. For cells that are diploid or contain multiple copies of the target gene dispersed in the genome, multiple rounds of transfection with additional selectable markers are required to remove all gene copies. Alternatively, stepwise selection with increasing concentrations of drug can sometimes lead to replacement of the second allelic copy of the target by the gene for the selectable marker, a process known as “loss of heterozygosity” [39,40].

If null mutants can be generated by either of the above methods, then the target is not essential for survival *for that particular life cycle stage, under those specific in vitro culture conditions*. Clearly, if the biochemical phenotype can be predicted, then null mutants can be obtained by nutritional rescue with an appropriate supplement in the medium. For example, ornithine decarboxylase deficiency can be rescued by putrescine [41,42] or thymidylate synthase deficiency rescued by thymidine [43]. Since these culture conditions are *not* identical to the physiological milieu *in vivo*, where feasible, it is important to demonstrate that transgenic parasites in the correct life-cycle stage are not infectious in an appropriate animal model [44-46]. Unfortunately, this is rarely done.

For essential genes, multiple rounds of transfection either fail to generate any cell-lines that are resistant to the drugs used for selection or evade gene replacement by various mechanisms. Escape mechanisms include: insertion of the selectable marker into another part of the genome; increase in chromosome number (e.g. aneuploidy or tetraploidy) [47] or compensatory genetic mutations [48]. These “escape mutants” can be regarded as suggestive of essentiality [47], but should be backed up with additional studies such as nutritional rescue (see above) or genetic rescue experiments. In the latter case, this is achieved by insertion of another copy of the target (sometimes from a related species) either on an episomal vector or at another chromosomal locus [33-36]. Although this method is widely applicable, unfortunately, this reveals no information as to precisely what level of enzyme activity is compatible with growth or survival and consequently what level of inhibition has to be achieved by drug treatment. Where possible, inducible or repressible gene-expression system(s), such as the tetracycline-inducible systems for trypanosomes should be used instead [49-51].

At the RNA level, target levels can be knocked down by RNA interference (RNAi) by expression of double-stranded RNA (dsRNA) [52]. This method is only applicable at present for African trypanosomes [53] and possibly one species of leishmania parasite [53,54]. Inducible expression of dsRNA is preferable to constitutive expression, since transgenic organisms lacking an essential biochemical component generally cannot be selected – only “escape mutants” can be recovered making interpretation difficult. However, even with inducible systems, insufficient expression of dsRNA may fail to knock down target expression to levels required to reveal a phenotype. Thus a “negative” result in the absence of careful phenotypic characterization of target expression has little value. A “positive” result, where growth inhibition correlates with decreased target production, can be quite helpful. However, even positive results can be problematic, if RNAi causes “off-target” effects. To circumvent this concern, it is sometimes possible to rescue the RNA phenotype by expression of a gene from another species that differs sufficiently at the nucleotide level to be refractory to RNAi, as was recently demonstrated using *L. major* spermidine synthase expressed in *T. brucei,* [55].

As can be seen from the above discussion target knockout or knockdown is not without its limitations, emphasising the need for both chemical and genetic evidence to increase confidence that a putative target is both essential and druggable. When specific inhibitors are available then it is possible to employ a hybrid approach. Here, modulation of target levels through under- or over-expression can be used in conjunction with specific inhibitors to demonstrate on-target activity in intact cells through a corresponding shift in cell potency [44,56].

## DRUGGABILITY AND DRUG LIKENESS

In the 1990’s a high percentage of compounds entering clinical development failed for poor pharmacokinetic (PK) properties. Consequently, the pharmaceutical industry adapted to address PK a lot earlier in the drug discovery process. The result has been dramatic, and by the turn of the century, attrition had been significantly reduced in the clinic due to adverse PK and poor bioavailability [57].

In the search for understanding compound attrition due to poor PK, the “rule of 5”, also known as “Lipinski rules” [58], was introduced, and compounds that adhere to these rules were coined as “druggable” or “drug-like”. More recently, the terms “druggable protein” and “druggable genome” have been used in the context of protein targets that can bind drug-like compounds [59] such that “druggability” is the likelihood of being able to modulate a protein target with a small drug-like compound.

Traditionally, the selection of targets for drug discovery rarely included an assessment of the likelihood of discovering drug-like ligands [5]. This omission has contributed to the failure of many screening campaigns [60], as the binding sites of many targets are too large or too small, too polar or lack sufficiently deep binding pockets to potently bind drug-like compounds. It is therefore crucial that any new drug discovery project target is druggable and consequently of higher probability of successfully progressing from hit to lead.

There has been much debate about what proportion of the human genome is druggable, currently estimated at 8-12% of all genes [59]. This statistic is currently unknown for pathogens, but is likely to be similar. However, this raises the importance of being able to predict how druggable a novel pathogen target is in early drug discovery. There are a number of well documented approaches available to identify potential druggable targets. A target’s druggability is usually estimated by classifying it with known gene families that have been successfully targeted with small drug-like compounds. This assumes that if one member of a gene family binds drug-like compounds, other members will do so, as binding-site architecture is generally conserved between gene family members. More recently however, newer approaches to predicting druggability have been reported. Notably, Hajduk and co-workers [61], using nuclear magnetic resonance data on the interactions of 10,000 lead-like or fragment-like compounds with protein surfaces, developed druggability indices that can be used for the computational assessment of proteins with known structure. Likewise, Cheng and co-workers [62] have devised a mathematical model that uses structural information about a target's binding site to estimate druggability. These new paradigms of assessing the suitability of targets for entering hit discovery represent potentially exciting ways of reducing the current high rates of attrition.

However, as stated by Keller  [63], it has never been challenging to find inhibitors of proteins with questionable druggability. The real druggability challenge arrives when these leads have to be turned into orally bioavailable drug candidates that have suitable properties to successfully advance through clinical development.

The value of including physicochemical property guidelines [58,64] in the selection of leads was an important step forward in starting with a foundation of leads that are free from major liabilities that would later impede the accomplishment of a viable clinical candidate.

During lead optimisation substructures are added onto the lead to enhance target affinity and selectivity. Non-polar groups are added to enhance binding to lipophilic pockets and other groups added to increase hydrogen bonding with the binding site. This adds lipophilicity, molecular weight and hydrogen bonding to the lead and can be highly detrimental to the pharmacokinetic properties of the compound. Consequently drug candidates that are rule-of-5 compliant would be easier to find if libraries are lead-like, only containing compounds with lower molecular weight, lower lipophilicity, and fewer hydrogen bonds so that once optimised, the candidate compound is still drug-like.

There are limitations to the rule of 5 as this only applies to compounds that are delivered by the oral route. If the TPP allows an alternative route of administration other than oral, then there may be room for flexibility in physicochemical properties and appropriate adjustments to the initial drug-like properties of a lead factored in. Injectable compounds can be significantly heavier, more polar and more flexible than oral compounds [65]. Furthermore, an injectable compound should have higher solubility than an oral compound. Inhaled compounds can also be heavier, more polar and have a higher polar surface area than orally administered compounds [66]. In contrast the properties of topical compounds are similar to an oral compound [65].

Additionally, the site of action of the potential drug needs to be considered. If the compound is to be delivered by the oral route, but must also cross the blood-brain barrier to exert its effect, as for stage 2 HAT, additional considerations need to be made in further restricting the physicochemical properties of the initial lead-like compounds. For compounds required to cross the blood-brain barrier, restricted Lipinski rules have been adopted for selecting initial lead templates. Notably, a significantly lower polar surface area to that required for oral bioavailability [67] is required.

The properties of the final compound also have to comply with TPP requirements such as drug stability and cost of goods. For such diseases, both the candidate compound and the formulated form of the drug need to be stable for extended periods, 2-3 years, under tropical conditions, as it is undesirable and unpractical in many situations to have temperature controlled storage systems. Therefore the compound classes developed need to be devoid of functionality that is inherently unstable, such as those commonly found in irreversible inhibitors. In addition the formulations used to deliver the drugs need to be simple and not to require special storage conditions.

For compounds to comply with strict restrictions on the cost of treatment, the candidate compounds in turn have to be to cheap to make using low cost intermediates and high yielding, straightforward chemistry involving a limited number of synthetic steps. Therefore, early judgements can be made when assessing the targets and hits from screens, whether they will deliver compounds of low complexity and cost.

Integrating the concepts of drug likeness and druggability for both target selection and lead selection into the drug discovery process should ultimately reduce the current high rates of attrition in the hit to lead and lead optimisation process.

## RESISTANCE POTENTIAL

Development of resistance during the use of antimicrobials is almost inevitable and therefore, strategies must be adopted in clinical use of antimicrobials to deal with this. One of the most widely used strategies is combination therapy, using compounds targeting different pathways or with different patterns of mutations preventing cross-resistance if acting on the same target [13,68-70]. Despite this, many anti-infective programmes take place against a background of the need to replace existing drugs due to the emergence of resistance. This need will be reflected in the TPP. Thus, the targets investigated need to be assessed for their ability to avoid cross resistance with the current therapies. Indeed, an advantage would be gained if the parasite biology suggested the target under consideration could be synergistic with the currently used drugs.

When assessing potential new targets, the underlying biology would suggest that in some cases there may be facile routes of resistance, which will rapidly reduce the clinical effectiveness of any particular drug. Identifying these mechanisms during target validation is important, as it will reflect on the validity of a target and maybe preclude the development of the target into drug discovery.

Resistance can arise from a number of different sources: point mutation of the target enzyme; over-expression of the target enzyme; efflux of the drug from parasites via transporters; reduced drug uptake through transporters; induced metabolism of the drug; and use of by-pass pathways. It is also possible that a pathogen can adapt to living in the presence of a chemotherapeutic agent, for example through increased uptake of metabolites downstream of the point of inhibition in a pathway.

Identification of potential mechanisms of resistance can be difficult to achieve. However, analysis of the relevant genome can help to identify some mechanisms, such as potential by-pass mechanisms and orthologous proteins.

Therefore, when selecting a drug target, potential mechanisms of resistance need to be considered. It has to be noted though, that development of resistance in a laboratory setting does not necessarily mirror what will happen in a clinical setting.

## TOXICITY PROFILES

One of the key aspects covered within a TPP is the acceptable toxicity profile of the prospective drug candidate. Therefore, when selecting a target for a drug discovery programme, an important consideration is the potential of modulation of the target or the compounds required to modulate the target to give rise to toxicity. Toxicity can arise via a number of sources, such as inherent toxicity due to modulation of a host target, modulation of host targets that are close homologues of the parasite target under consideration, modulation of unrelated human enzymes or receptors, and toxicity due to the structure of a drug or the metabolite of a drug.

The level of acceptable toxicity for any treatment will depend on a number of factors including: the severity of the disease; the target patient population; the existence of other treatments; the clinical setting; and the length of proposed treatment. Thus for a life-threatening disease, such as certain cancers for which there is no other treatment, then a higher level of toxicity could be acceptable, than for a non life-threatening disease such as pain management, for which other treatments exist.

Although many neglected diseases are life threatening, the target population will be made up of a significant proportion of women of childbearing age and children. In addition, the vast majority of patients have very limited access to sophisticated medical support, therefore complex monitoring of drug levels or toxicity indicators is impractical; consequently, safety profiles need to reflect these special needs.

In terms of assessing toxicity, the inherent toxicity of a molecular target can be minimised in the case of anti-microbial chemotherapy by targeting parasite specific enzymes and processes which have no analogues in human. However, many of these targets have no history of drug discovery and some are of dubious druggability. Therefore, many of the most promising targets have human homologues requiring in most instances for the compounds to be highly selective for the microbial target over a corresponding human enzyme. For many targets there will be sufficient differences to derive selectivity. Conversely, where there is very high structural similarity between some pathogen targets and their human homologues, inhibition of such a target is likely to give rise to toxic effects. In consequence this particular molecular target is very unlikely to satisfy a TPP, as it will be very difficult to obtain selective compounds, unless the differences of the function in human and parasite cells allowed selectivity at the level of the biology of the system.

For example, a number of anti-proliferative kinase inhibitors have been developed as anti-cancer agents, which could have interesting anti-proliferative activity against parasite kinases. However, by their very nature these compounds are likely to be teratogenic or foetotoxic precluding their use for women, as it would be difficult to test for pregnancy in a neglected diseases clinical setting. Therefore, parasite kinase inhibitors will need to be either designed and/or profiled to ensure selectivity over anti-proliferative human kinases. The development toxicology package would need to be designed to include reproductive toxicity studies at an early stage to ensure the compounds are not teratogenic or foetotoxic.

At the target assessment stage the type of compounds required to inhibit the proposed target needs to be considered for their toxicity potential. Prediction of toxicity using *in silico* methodology is still in its relative infancy. Therefore during the drug discovery process, potential liabilities should be identified as early as possible through appropriate assays, allowing go/no-go decisions to be made on particular chemical series and targets. During early stage discovery, removal of compounds with functional groups associated with toxicity (e.g. Michael acceptors) and testing of key compounds for liabilities, such as potentially fatal cardiotoxicity due to inhibition of the hERG potassium channel, should help to reduce compound-associated toxicity. Irreversible inhibitors are often proposed as modulators of poorly druggable targets, such as certain proteases. However, the intrinsic reactivity of these compounds make them more likely to be toxic either through direct interaction with host enzymes or through reaction with protein residues, which can induce immunogenicity. Again these compounds could be toxic to rapidly dividing cells including those of a foetus. If compounds of this type are developed the toxicity programme would need to reflect the increased chance of such toxicity.

The development programme of drug candidates, including the toxicology studies, needs to take into account the differences seen in drug metabolism and transport across ethnic groups, known as pharmacogenetics [71]. For example, there is variability in the expression levels and isoform expression of cytochrome P450s across different ethnic populations. This variability can cause either a decrease or increase in exposure to an experimental compound resulting in a loss of activity, or a potential increase in toxicity respectively. In addition, differing expression of P450 isoforms could change the metabolic routes of compounds causing the production of metabolites not accounted for in toxicity studies or cause unexpected drug-drug interactions in cases where patients are likely to be on several medicines at the same time, due to difference in P450 inhibition profiles. Therefore, it is important the pre-clinical and clinical studies reflect the ethnicity of the intended patient population.

## CONCLUSION

The landscape of neglected diseases drug discovery is beginning to change dramatically with the establishment of credible and productive PPP and the rekindling of interest from the Pharma sector. These changes in turn are encouraging the development of new collaborations between academic groups, PPPs and Pharma partners.

Despite these encouraging developments, there hangs the spectre of the high attrition rates experienced by all sectors involved in drug discovery. Therefore, in this still under resourced area of drug discovery, there needs to great clarity around the aims and goals of the mission and clear decision making associated with each project. To this end Therapeutic Product Profiles are coming to the fore, allowing the unmet medical need and the properties required of the clinical drug and its usage to be clearly defined. Using this information, the criteria for transition from each stage of the drug discovery process can be clearly defined, even back to making assessments on whether a target could eventually deliver candidates which could satisfy the TPP.

It is hoped that this review will encourage scientists to use TPPs to help assess potential targets, and their progress toward the ultimate goal of discovering therapies to alleviate the suffering of many of the most under-privileged people in the world. In addition we would urge those studying potential novel targets to generate the appropriate information as a matter of course to enable the targets to be assessed for suitability for drug discovery projects. In doing so, a robust pipeline of potential anti-parasitic drug targets can be built for the future.

## Figures and Tables

**Table 1:** Parasitic Disease Target Product Profiles for Human African Trypanosomiasis and Malaria^a^

**General desired features for a drug include:** Therapeutic area Spectrum of activity (e.g. active against all species including drug resistant isolates) Target population (e.g. pregnant women and children) Dose, frequency and route of administration (e.g. once a day, oral route) Safety and efficacy (better than existing treatments) Toxicity (minimal side effects, better than existing treatments) Potential for use in drug combinations (minimise emergence of resistance) Few contraindications (e.g. minimal drug-drug interactions; suitable for use in HIV/AIDS or TB co-infections) Low potential of developing parasite resistance Stability under tropical conditions (i.e. > 2 years shelf life at 40 °C and 75% relative humidity) Cost of goods (i.e. equivalent to or cheaper than existing treatments)
**A TPP for uncomplicated falciparum malaria includes:** Oral (ideally once per day for not more than three days) Low cost of goods (~US$1 per full course of treatment) Effective against drug-resistant parasites (e.g. those that have developed resistance to chloroquine or Fansidar) Fast acting and curative within three days Potential for combination with other agents Paediatric formulation should be available Stable under tropical conditions
**A TPP for human African trypanosomiasis (HAT) includes:** Active against *T.b.gambiense* and *T.b.rhodesiense* Active against melarsoprol refractory strains Efficacy against early and late-stage disease desirable Formulation (oral against early stage desirable; parenteral against late stage acceptable) Curative in 14 days (late stage) or less (early stage) Cost less than current treatment for early stage disease ($100-140) Safe in pregnancy Stable under tropical conditions

a Additional TPPs for neglected diseases such as schistosomiasis are available elsewhere [3]. Further details of the various TPPs for other malaria indications are available from Medicines for Malaria Venture (MMV) website (http://www.mmv.org/). Additional desirable and acceptable criteria for visceral leishmaniasis and Chagas’ disease are available from Drugs for Neglected Diseases initiative (http://www.dndi.org/)

**Table 2:** Traffic Light Definitions for Target Assessment Used in the Drug Discovery Unit at the University of Dundee.

Criterion	Red	Amber	Green
Target validation	No or weak evidence that the target is essential for growth or survival	Either genetic or chemical evidence that target is essential for growth or survival a	Genetic and chemical evidence that target is essential for growth or survival a
Druggability	No drug-like inhibitors are known and active site of target is not druggable	Drug-like inhibitors are known or active site potentially druggable	Drug-like inhibitors are known and druggable active site (i.e. clinical precedent within the target family)
Assay feasibility	No *in vitro* assay developed and / or significant problems with reagents (cost or supply)	*In vitro* assay exists, development into plate format feasible, but not achieved	Assay ready in plate format and protein supply assured within appropriate timelines
Toxicity	Human homologue present and little or no structural or chemical evidence that selective inhibition is possible	Human homologue present, but some structural or chemical evidence that selective inhibition is possible	No human homologue present or human homologue known to be non-essential
Resistance potential	Target has multiple gene copies or isoforms within same species and is subject to escape from inhibition b	Target has isoforms within same species or may be subject to escape from inhibition b	Target has no known isoforms within same species and is not subject to escape from inhibition b
Structural information	No structure of target or closely related homologue	Structure without ligand available and / or poor resolution (> 2.3 Å) or opportunity to build a good homology model	Ligand bound structure of target (or ligand in closely related homologue) available at high resolution (< 2.3 Å)

a See Table 3 for the relative merits of genetic and chemical validation.

b Possible resistance mechanisms include: accumulation of substrate that could reverse inhibition; or target can be deleted, readily modified by point mutation, readily amplified or bypassed.

**Table 3:** Strengths and Weaknesses of Different Target Validation Methods (Modified From [11])

Method	Strengths	Weaknesses
Chemical validation	Addresses the key druggability issues of cell permeability (*in vitro* whole cell assays); selective toxicity and drug metabolism (*in vivo* animal models); safety and efficacy (clinical) Identifies non-protein targets Identifies pro-drugs and compounds acting by lethal synthesis	Highly specific inhibitors frequently not available Lack of specificity for target resulting in poor structure-activity-relationships (SAR) Variable cellular pharmacokinetics can cause poor SAR Correlation between target inhibition and predicted molecular or biochemical phenotype sometimes difficult to demonstrate in vitro or in vivo
All genetic validation	Many complete genomes available Suitable for genes of unknown or uncertain function	Cannot identify non-gene targets (e.g. haemozoin) Does not address key druggability issues Does not identify drugs acting via lethal synthesis Does not distinguish between structural and catalytic requirement
Knockout methods	Definitive, “clean” phenotype Few or no off-target effects	Laborious (usually requires multiple transfections in diploid organisms) Null mutants for essential genes require genetic or nutritional rescue Multicopy genes can be problematic Compensatory (suppressor) mutations can occur
RNA interference (RNAi)	Rapid and easy to perform Suitable for multicopy gene families	Not possible in many parasite species No phenotype due to insufficient silencing Off-target effects due to unintentional silencing “Escape” mutants with essential genes
